# Comparative assessment of clinical rating scales in Wilson’s disease

**DOI:** 10.1186/s12883-017-0921-3

**Published:** 2017-07-21

**Authors:** Hanna M. Volpert, Jan Pfeiffenberger, Jan B. Gröner, Wolfgang Stremmel, Daniel N. Gotthardt, Mark Schäfer, Karl Heinz Weiss, Markus Weiler

**Affiliations:** 1 Department of Internal Medicine IV, Heidelberg University Hospital, Im Neuenheimer Feld 410, Heidelberg, Germany; 2 Department of Neurology, Heidelberg University Hospital, Im Neuenheimer Feld 400, 69120 Heidelberg, Germany; 3 Department of Internal Medicine I, Heidelberg University Hospital, Im Neuenheimer Feld 410, Heidelberg, Germany

**Keywords:** Copper metabolism, German network of hereditary movement disorders (GeNeMove), Global assessment scale for Wilson’s disease (GAS for WD), Unified Wilson’s disease rating scale (UWDRS), Wilson disease

## Abstract

**Background:**

Wilson’s disease (WD) is an autosomal recessive disorder of copper metabolism resulting in multifaceted neurological, hepatic, and psychiatric symptoms. The objective of the study was to comparatively assess two clinical rating scales for WD, the Unified Wilson’s Disease Rating Scale (UWDRS) and the Global Assessment Scale for Wilson’s disease (GAS for WD), and to test the feasibility of the patient reported part of the UWDRS neurological subscale (termed the “minimal UWDRS”).

**Methods:**

In this prospective, monocentric, cross-sectional study, 65 patients (median age 35 [range: 15–62] years; 33 female, 32 male) with treated WD were scored according to the two rating scales.

**Results:**

The UWDRS neurological subscore correlated with the GAS for WD Tier 2 score (*r* = 0.80; *p* < 0.001). Correlations of the UWDRS hepatic subscore and the GAS for WD Tier 1 score with both the Model for End Stage Liver Disease (MELD) score (*r* = 0.44/*r* = 0.28; *p* < 0.001/*p* = 0.027) and the Child-Pugh score (*r* = 0.32/*r* = 0.12; *p* = 0.015/*p* = 0.376) were weak. The “minimal UWDRS” score significantly correlated with the UWDRS total score (*r* = 0.86), the UWDRS neurological subscore (*r* = 0.89), and the GAS for WD Tier 2 score (*r* = 0.86).

**Conclusions:**

The UWDRS neurological and psychiatric subscales and the GAS for WD Tier 2 score are valuable tools for the clinical assessment of WD patients. The “minimal UWDRS” is a practical prescreening tool outside scientific trials.

**Electronic supplementary material:**

The online version of this article (doi:10.1186/s12883-017-0921-3) contains supplementary material, which is available to authorized users.

## Background

Wilson’s disease (WD) is an autosomal recessive disorder caused by mutations in the *ATP7B* gene leading to excessive copper overload, predominantly in the liver and the brain [1, 2]. The severity of the disease varies considerably between patients, and it remains unclear why some patients have hepatic symptoms while others develop neurological, psychiatric, or combined symptomatology [3–5]. Hepatic features range from asymptomatic patients with slightly elevated liver enzymes to liver cirrhosis or acute hepatic failure [6]. Neurological symptoms are usually characterized by multiple motor impairments characteristic for dysfunctions of the basal ganglia and the cerebellum. This results in various neurological symptoms including rigidity, tremor, dyskinesia, dystonia, ataxia, chorea, dysarthria, dysphagia, or excessive salivation [6–10]. Psychiatric symptoms are diverse and may include concentration difficulties, attention disorders, behavioral abnormalities with alterations of personality, depression, and psychosis [11–14]. The severity of the symptoms clearly determines the extent to which a patient is restricted in their activities of daily living (ADL).

To prevent progression of the disease and lethal outcomes, lifelong therapy pursuing a negative copper balance is imperative; hence, underlining the importance of an early diagnosis and an individually adapted therapy [15]. However, neurological worsening in WD patients following initiation of penicillamine standard therapy is a major concern [16]. Given the multifaceted symptomatology in WD, a comprehensive, standardized, and practical clinical rating scale is indispensable to monitor individual therapeutic effects and side effects in daily practice and to use as a valid end point in clinical trials testing therapies.

Currently, there are two clinical rating scales for WD: the Unified Wilson’s Disease Rating Scale (UWDRS) and the Global Assessment Scale for Wilson’s Disease (GAS for WD). The UWDRS has neurological, hepatic, and psychiatric subscales. The neurological subscale of the UWDRS was developed in collaboration with the European Network, EuroWilson, and the German Network of Hereditary Movement Disorders (GeNeMove). The first WD rating scale reflecting the extent of neurological impairment was reported by Czlonkowska et al. in 2007 [17]. It was extended by hepatic and psychiatric subscales that were evaluated in total by Leinweber et al. in 2008 [18]. The GAS for WD, considerably shorter than the UWDRS, is comprised of two tiers scoring global disability (Tier 1) and neurological dysfunction (Tier 2) and was proposed by Aggarwal et al. in 2009 [19]. Potential difficulties that have arisen from both rating scales in daily practice are the relatively long duration of the complete clinical assessment and the formal requirement of two to three different medical specialists (i.e., neurology, gastroenterology, and psychiatry).

So far the UWDRS and the GAS for WD have not been directly compared, and it remains unclear which rating scale may be superior. Hence, the goal of the present study is to evaluate the UWDRS and the GAS for WD in routine clinical practice and to propose a less time consuming patient reported prescreening tool for use outside scientific trials, the “minimal UWDRS”.

## Methods

### Patients

This prospective, monocentric, cross-sectional study was carried out between 2014 and 2015. The ethics committee at the University of Heidelberg (Heidelberg, Germany) approved the study. A total of 65 patients with treated WD were evaluated according to the UWDRS and the GAS for WD. Patients older than 14 years of age who had been diagnosed with WD according to the diagnostic and phenotypic classification of WD [2] by the Department of Gastroenterology at Heidelberg University Hospital were enrolled after giving written informed consent. For patients who were under 18 years of age, written informed consent was obtained from the parents or the legal guardians. Patient related information including gender, age at assessment, age at the time of diagnosis, initial mode of manifestation, treatment at assessment, and clinical parameters were collected.

### Clinical evaluation of the UWDRS and the GAS for WD

The full UWDRS (maximum score 320 points) consists of three subscales representing three main features of clinical manifestation in WD: a neurological subscale (27 items, 208 points), a hepatic subscale (9 items, 36 points), and a psychiatric subscale (19 items, 76 points). Of the 55 items in total, the patient has to answer 26 questions, whereas 29 items need to be scored by the observer. Each item is scored on an ascending five point scale (0 points “no symptoms” and 4 points “worst characteristic possible”) [17, 18].

The GAS for WD contains two parts. Tier 1 scores global disability in four areas: liver, cognition and behavior, motor, and osseomuscular (with an ascending six point scale (0–5); the scores of each item are not summed up). Tier 2 scores neurological dysfunction and consists of 14 items with an ascending five point scale (0–4). The items of Tier 2 are summed for a possible maximum of 56 points [19]. All clinical assessments were performed by a resident in internal medicine (H. M. Volpert) supervised by both an attending hepatologist (K. H. Weiss) and an attending neurologist (M. Weiler). Disagreements between the investigators were resolved by discussion.

### The “minimal UWDRS”

The first nine items of the UWDRS neurological subscale were selected to develop a minimal neurological subscale, newly determined as the “minimal UWDRS.” Since this reduced rating scale exclusively represented a questionnaire whose items are reported by the patient or their family (usually referring to the previous two to four weeks), the resulting score can be assessed before the consultation with the treating physician. The selected items are: item 1 “mobility”, item 2 “falling”, item 3 “salivation”, item 4 “swallowing”, item 5 “feeding”, item 6 “dressing”, item 7 “taking a bath or shower”, item 8 “grooming”, and item 9 “toilet use”. Apart from items 3 and 4, all other questions assess the level of independence for ADL.

### Statistical analysis

#### Comparison of the UWDRS with the GAS for WD

UWDRS and GAS for WD were compared by correlating the scores of the rating scales and subscales in scatter diagrams and by calculating the Pearson’s correlation coefficient. Correlation coefficients were interpreted as weak (*r* < 0.5), moderate (*r* = 0.5–0.79), and strong (*r* ≥ 0.80) [20]. To validate the UWDRS hepatic subscore and the GAS for WD Tier 1 “liver” domain, these items were each correlated with the Model for End Stage Liver Disease (MELD) score and the Child-Pugh score [21, 22]. The frequencies of scoring 1, 2, 3, or 4 points were calculated as well as the percentage of scoring >0 points for each item. Internal consistencies were evaluated using Cronbach’s alpha (values of ≥0.7 were interpreted as “good”, and ≥0.9 were interpreted as “very good”) [18]. Cronbach’s alpha was used to compare our results with the results of previous studies that evaluated the two clinical rating scales [18, 19]. In addition, single item total score correlations were assessed.

#### Evaluation of the “minimal UWDRS”

The score of the newly developed “minimal UWDRS” was correlated with the scores of the UWDRS and the GAS for WD including their neurological subscores. A *p* value <0.05 was considered statistically significant. For statistical analysis, SPSS™ (version 20.0, IBM Germany, Ehningen) was used.

## Results

### Descriptive analysis

Table 1 gives an overview of the clinical characteristics of the 65 WD patients examined. Patients that presented with hepatic symptoms as the initial mode of manifestation totalled 70.8%. Table 2 shows the results of the UWDRS and the GAS for WD both in general and in dependence from the initial mode of manifestation (i.e., hepatic, neurological, combined hepatic/neurological, and asymptomatic). As indicated by the relatively low medians, patients with severe WD symptoms were rare throughout the entire study. When patients were assessed depending on their initial mode of manifestation, the UWDRS total score and the UWDRS neurological subscore differed significantly (*p* values 0.026 and 0.012, respectively), which reflected the neurological disease burden in this subgroup. Additional file 1: Table S1 shows significantly differing results of the UWDRS and the GAS for WD when comparing clinical subgroups with respect to gender, Kayser-Fleischer rings (KFR) at assessment, and liver cirrhosis at the time of diagnosis. The prevalence of subscores of each item including the respective single item total score correlations are listed in the supporting data (Additional file 1: Figure S1).

**Table 1:** Clinical characteristics of the 65 examined patients with WD

	N	Percentage	Median	Range
Gender				
Female	33	50.8		
Male	32	49.2		
Age at assessment (yr)				
All			35	15–62
Female			40	15–62
Male			29.5	16–62
Age at onset of symptoms (yr)			16	1–47
Age at time of diagnosis (yr)			17	3–54
Initial mode of manifestation				
Hepatic	46	70.8		
Hepatic + neurological	10	15.4		
Neurological	6	9.2		
Asymptomatic	3	4.6		
Liver cirrhosis at time of diagnosis	21	32.3		
*By initial mode of manifestation*				
Hepatic [*n* = 46]	15	32.6		
Hepatic + neurological [*n* = 10]	6	60.0		
Liver status at assessment				
MELD score [missing data *n* = 4]			7.3	6–17
Child-Pugh score [missing data *n* = 7]			5	5–11
KFR at time of diagnosis [missing data *n* = 8]	28	49.1		
KFR at assessment	19	29.2		
Treatment at assessment in all patients (*n* = 65)				
D-Penicillamine	38	58.5		
Trientine	16	24.6		
Zinc	7	10.8		
D-Penicillamine + zinc	2	3.1		
Trientine + zinc	2	3.1		
Treatment at assessment in neurologically symptomatic patients (*n* = 16)				
Chelating agents	13	81.2		
Zinc with or without chelating agent	3	18.8		
Duration of treatment (yr)			15	0.4–47

*Abbreviations: KFR* Kayser-Fleischer ring, *pts.* patients, *yr.* years

**Table 2:** Overview of the UWDRS and GAS for WD Tier 2 (sub)scores

	Total score median (range)				*P* value
UWDRS total score	10 (0–97)				
UWDRS neurological subscore	5 (0–74)				
UWDRS hepatic subscore	2 (0–13)				
UWDRS psychiatric subscore	1 (0–26)				
GAS for WD Tier 2 score	3 (0–24)				
	Initial mode of manifestation				
	Hepatic	Hepatic + neurological	Neurological	Asymptomatic	
UWDRS total score	9 (0–55)	32.5 (2–97)	18.5 (5–76)	4 (3–5)	0.026*
UWDRS neurological subscore	6 (0–49)	21.5 (1–66)	14 (3–74)	4 (2–4)	0.012*
UWDRS hepatic subscore	2 (0–13)	2.5 (0–12)	1 (0–5)	1 (0–1)	0.546
UWDRS psychiatric subscore	1 (0–12)	1.5 (0–26)	2 (2–7)	0 (0–0)	0.081
GAS for WD Tier 2 score	2 (0–24)	5 (0–23)	4.5 (1–11)	2 (1–3)	0.099

*, *P* value statistically significant

### Correlations of the UWDRS, GAS for WD, and “minimal UWDRS”

**Fig. 1:**
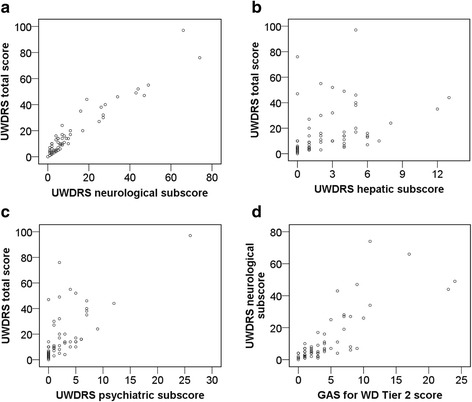
**a** Correlation of the UWDRS total score with the UWDRS neurological subscore. **b** Correlation of the UWDRS total score with the UWDRS hepatic subscore. **c** Correlation of the UWDRS total score with the UWDRS psychiatric subscore. **d** Correlation of the UWDRS neurological subscore with the GAS for WD Tier 2 score

The correlation of the UWDRS total score with the UWDRS neurological subscore is shown in Fig. 1a (correlation coefficient of *r* = 0.96; level of significance of r: *p* < 0.001). The UWDRS total score correlated with the UWDRS hepatic subscore to a lower extent (correlation coefficient of *r* = 0.37; level of significance of r: *p* = 0.003; Fig. 1b) than the UWDRS total score with the UWDRS psychiatric subscore (correlation coefficient of *r* = 0.83; level of significance of r: *p* < 0.001; Fig. 1c). Moreover, the UWDRS neurological subscore correlated with the GAS for WD Tier 2 score (correlation coefficient of *r* = 0.80; level of significance of r: *p* < 0.001; Fig. 1d).

**Fig. 2:**
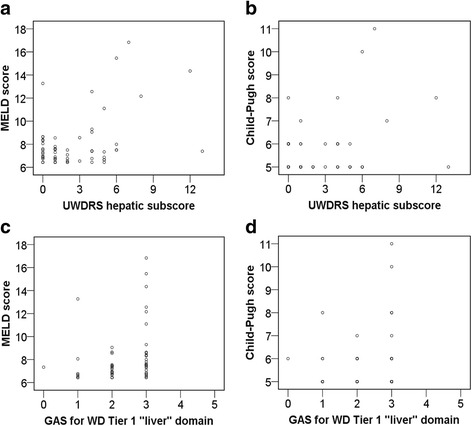
**a** Correlation of the MELD score with the UWDRS hepatic subscore. **b** Correlation of the Child-Pugh score with the UWDRS hepatic subscore. **c** Correlation of the MELD score with the GAS for WD Tier 1 “liver” domain. **d** Correlation of the Child-Pugh score with the GAS for WD Tier 1 “liver” domain

Figure 2 depicts the correlations of the UWDRS hepatic subscore and the score of the GAS for WD Tier 1 “liver” domain, respectively, with each the MELD and the Child-Pugh score. The UWDRS hepatic subscore correlated weakly with both the MELD score (correlation coefficient of *r* = 0.44; level of significance of r: *p* < 0.001; Fig. 2a) and the Child-Pugh score (correlation coefficient of *r* = 0.32; level of significance of r: *p* = 0.015; Fig. 2b). For the score of the GAS for WD Tier 1 “liver” domain, results were similar: correlations with each the MELD score (correlation coefficient of *r* = 0.28; level of significance of r: *p* = 0.027; Fig. 2c) and the Child-Pugh score (correlation coefficient of *r* = 0.12; level of significance of r: *p* = 0.376; Fig. 2d) were weak.

**Fig. 3:**
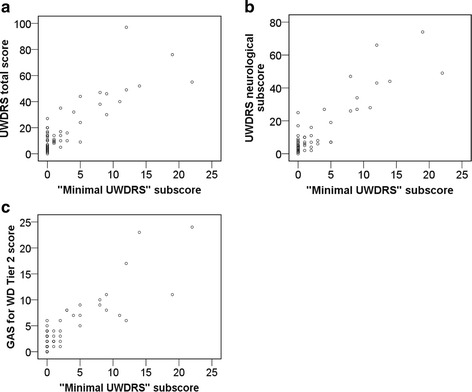
**a** Correlation of the UWDRS total score with the UWDRS minimal neurological (“minimal UWDRS”) subscore. **b** Correlation of the UWDRS neurological subscore with the UWDRS minimal neurological (“minimal UWDRS”) subscore. **c** Correlation of the GAS for WD Tier 2 score with the UWDRS minimal neurological (“minimal UWDRS”) subscore

The “minimal UWDRS” score significantly correlated with each the UWDRS total score (*r* = 0.86; Fig. 3a), the UWDRS neurological subscore (*r* = 0.89; Fig. 3b), and the GAS for WD Tier 2 score (*r* = 0.86; Fig. 3c).

### Single item total score correlation and Cronbach’s alpha

Single item total score correlations are listed in the supporting data (Additional file 1: Figure S1, right). Cronbach’s alpha for the total UWDRS was 0.95, for the UWDRS neurological subscale it was 0.96, for the UWDRS hepatic subscale it was 0.65, and for the psychiatric subscale it was 0.83. No item could be deleted in order to improve Cronbach’s alpha for the UWDRS neurological subscale. After omitting item 30 “fecal blood”, Cronbach’s alpha for the UWDRS hepatic subscale changed to 0.66. The three items 39a “sexual interest increased”, 50 “voice/noise level”, and 52b “mood depressed” were left out to improve the internal consistency of the UWDRS psychiatric subscale to 0.84. The internal consistency of the GAS for WD Tier 2 score (Cronbach’s alpha of 0.82) was slightly lower than the one for the UWDRS neurological subscale. After omitting item 13 “KFR”, Cronbach’s alpha improved to 0.84.

## Discussion

In the clinical management of WD, thorough clinical monitoring including early detection of potential medical side effects and therapy failure is crucial to improve the patient’s motivation and adherence [23]. Therefore, liver function tests and the calculation of MELD and Child-Pugh scores on an every three months basis are recommended [6, 21, 24]. During the initial phase of treatment, the modified Nazer score is a reliable tool to identify patients at risk for hepatic treatment failure requiring liver transplantation [25, 26].

Additionally, ultrasound of the liver visualizes the status of cirrhotic reorganization. However, an adequate assessment of neurological impairment has long been neglected due to the lack of a suitable tool for evaluation. Standardized neurological assessment allows for both intra- and inter-individual comparison of symptoms, which improves follow up and adds the ability of quantifying the severity of neurological symptoms. Especially for clinical trials on effects and side effects of medical treatments in WD, neurological rating scales are invaluable [27, 28]. This study presents the first systematic comparison of two clinical rating scales previously developed for WD, the UWDRS and the GAS for WD, and evaluates their feasibility in routine clinical practice. Additionally, we introduce the patient reported part of the UWDRS neurological subscale, the “minimal UWDRS”, as a handy and time saving prescreening tool for the assessment of WD patients in routine clinical practice outside scientific trials.

The significant differences in the UWDRS total score and the UWDRS neurological subscore observed in our study show that the results differ depending on the initial mode of manifestation (Table 2). The strong correlation of the UWDRS total score with the UWDRS neurological subscore (Fig. 1a) demonstrates that the UWDRS neurological subscale represents the UWDRS total scale very well. As the UWDRS neurological subscore can total 208 points at maximum, or 65% of the total score, one reason for the strong correlation is that the UWDRS neurological subscore largely represents the UWDRS total score. In contrast, the correlation of the UWDRS total score with the UWDRS hepatic subscore is weak (Fig. 1b). Hence, the UWDRS hepatic scale does not reflect chronic compensated liver disease, which is common in WD [6]. The moderate correlation of the UWDRS total score with the UWDRS psychiatric score (Fig. 1c) reveals that patients with severe neurological or hepatic symptoms do not necessarily have pronounced concomitant psychiatric symptoms [14, 29]. More than 20% of the patients in the study were found to have failing memory (item 40), concentration difficulties (item 41), and reduced social contact (item 42), all being psychiatric disabilities frequently seen in patients with WD [8, 30, 31]. A brief global psychiatric assessment as provided by the UWDRS psychiatric subscale is doubtlessly inappropriate to reflect such disorders. Nevertheless, it can be supportive in identifying patients with WD who would benefit from referral to a psychiatrist for further exploration.

The UWDRS neurological subscore correlates strongly with the GAS for WD Tier 2 score (Fig. 1d). Since the UWDRS scale includes a larger number of neurology and psychiatry related items than the GAS for WD scale, it documents these disabilities in greater detail (with the caveat that GAS for WD includes Kayser-Fleischer rings as a feature, while the UWDRS does not). However, this comes at some cost. In routine clinical practice, the larger number of items in the neurological and psychiatric subscales of the UWDRS requires significantly more time to assess than the Tier 2 score of the GAS for WD. However, this may not be a concern in clinical trials, particularly in those that assess therapeutic effects when detailed documentation of a change in a single neurological symptom is inevitable [32]. More significantly, many of the detailed neurological signs in the UWDRS scale are difficult to reliably discern in practice, as is reflected in the low interrater reliability (i.e., small values for intraclass correlation coefficients [ICC] for some of the individual items of the scale) [18]. In contrast, all the individual items of the GAS for WD have ICC values in the high range (i.e., can be assessed reliably) [19]. Whether a larger number of detailed items or a smaller number of reliably assessable items are of greater benefit will depend on the goal and the design of the individual clinical trial, as well as the consistency in raters. For instance, a trial interested in a specific neurological feature (e.g., autonomic disturbances) that is included only in the UWDRS is probably preferable. Likewise, the GAS for WD may be more suitable for a longitudinal trial with the goal of assessing a change in item scores in individual patients over a long period of time involving many different raters, as it reduces rater induced variance.

The weak correlation of the UWDRS hepatic subscore with the MELD score (Fig. 2a) and the Child-Pugh score (Fig. 2b) demonstrates that the UWDRS hepatic subscore does not mirror the current liver status very well. The correlation of the GAS for WD Tier 1 “liver” domain with the MELD score (Fig. 2c) and the Child-Pugh score (Fig. 2d) is also weak (i.e., this item does not represent the liver status very well either). Furthermore, we could not reproduce the correlation coefficient of *r* = 0.65 with respect to the Tier 1 “liver” domain and the Child-Pugh score published by Aggarwal et al. in 2009 (in our study: *r* = 0.12). However as the Child-Pugh score, as well as the MELD score to a lower extent, might have a limitation in detecting significant changes of the hepatic function in clinically stable or non-cirrhotic liver patients, the most meaningful mode of evaluation for the hepatic system in a WD specific rating scale has yet to be established.

The “minimal UWDRS” score correlates strongly with the UWDRS total score (Fig. 3a), the UWDRS neurological subscore (Fig. 3b), and the GAS for WD Tier 2 score (Fig. 3c). Outside clinical trials, the “minimal UWDRS” is a convenient and time saving prescreening tool to document neurological impairments in WD patients. The neurological questionnaire can be handed to the patient in the waiting area before the medical appointment without the requirement of a neurologist. Consequently, the questionnaire will help to better structure the consultation as the patient would have already deliberated his actual complaints and would be more prepared for the physician’s questions. Besides, a short questionnaire, likely to be applied more frequently than an extensive neurological assessment, will support self reflection on disease symptoms and thus improve coping strategies as well as adherence to the treatment. Furthermore, the use of a questionnaire is more economical (after an initial detailed assessment), since it can be applied to figure out which impairments should be focused on and reevaluated at future follow up examinations. As seven out of nine items in the “minimal UWDRS” score the level of independence in the ADL, the physician will learn rapidly whether the neurological symptoms limit the patient’s ADL. Constrictively, the questionnaire does not interrogate the exact reasons underlying potential impairments of the ADL. A further limitation is the “minimal UWDRS” may not adequately capture the full breadth of neurological disability in patients with WD. For instance, the “minimal UWDRS” may miss mild limb dystonia or new onset Kayser-Fleischer rings, which can be early signs of noncompliance or inadequate treatment. Thus, the time saving will come at some cost to patient care.

Altogether, our study collective suffered from relatively mild to moderate WD symptoms (Table 2, Additional file 1: Figure S1). Hence, we can not use a single item total score correlation to select single items for a minimal neurological scale. Otherwise, a minimal neurological scale will be inappropriate to be applied for patients with severe WD symptoms. Internal consistencies of the UWDRS total score and its subscales, as well as the internal consistency of the GAS for WD neurological assessment, are all very similar to those reported by Leinweber et al. 2008 and Aggarwal et al. 2009 [18, 19]. In our study, Cronbach’s alpha for the UWDRS total score is 0.95 compared with 0.92 reported by Leinweber et al., the UWDRS neurological subscale is 0.95 compared with 0.94, the UWDRS hepatic subscale is 0.65 compared with 0.59, and the UWDRS psychiatric subscale is 0.83 compared with 0.76 [18]. Cronbach’s alpha for the GAS for WD Tier 2 in our study (0.82) and in the first report (0.89) is also similar [19]. In summary, we largely confirm the internal consistencies for both the UWDRS and the GAS for WD as reported by the previous two articles.

The high prevalence of the hepatic initial mode of manifestation (70.8% in this study vs. an average of 45% in the literature) observed in our study collective may be due to selection bias caused by the fact that all patients were recruited in a WD outpatient clinic in a Department of Gastroenterology. The low median MELD (7.3) and Child-Pugh (5) scores show that most patients suffered from compensated liver cirrhosis. In general, our study collective is only mildly to moderately affected, which is probably due to the median duration of treatment at study inclusion being 15 years (Table 1). Despite the relatively long median time of pretreatment, patients still were clinically impaired. This finding underlines the importance of future studies focusing on improvement of the medication utilized to treat WD.

## Conclusions

The main conclusions of our study are the following:

i) The UWDRS neurological and psychiatric subscales are valuable tools for a detailed assessment of neurological and psychiatric impairment in patients with WD.
ii) The GAS for WD Tier 2 can be considered as an alternative to gain an overview of the extent of neurological impairment.
iii) The UWDRS hepatic subscale, similar to the GAS for WD Tier 1, shows weak correlation to established hepatologic scores like MELD or Child-Pugh. This finding underlines the need for further assessment of liver specific rating scales in WD.
iv) We propose the patient reported “minimal UWDRS” (a nine item questionnaire) is a feasible, economical prescreening tool for the evaluation of the neurological status in WD patients with mild to moderate neurological symptoms.
v) Future studies are required to further evaluate the UWDRS, the GAS for WD, and the “minimal UWDRS” in WD patients with severe clinical symptoms.

## Additional file

Additional file 1: Figure S1. Prevalences of the single item scores. **Table S1.** UWDRS and GAS for WD Tier 2 (sub)scores depending on gender, KFR, and liver cirrhosis. (DOCX 506 kb)

## Abbreviations

ADL: Activities of daily living
GAS for WD: Global assessment scale for wilson’s disease
GeNeMove: German network of hereditary movement disorders
KFR: Kayser-fleischer rings
MELD score: Model of end stage liver disease score
UWDRS: Unified wilson’s disease rating scale
WD: Wilson’s disease
