# Supplementary material for: Comparative assessment of clinical rating scales in Wilson’s disease
Source: BMC Neurol. 2017 Jul 21;17:140. doi: 10.1186/s12883-017-0921-3 (PMC5521125; doi:10.1186/s12883-017-0921-3)
Supplement: Additional file 1: Figure S1. — Prevalences of the single item scores. Table S1. UWDRS and GAS for WD Tier 2 (sub)scores depending on gender, KFR, and liver cirrhosis. (DOCX 506 kb) [file 12883_2017_921_MOESM1_ESM.docx]

Supplementary Material

**Figure S1.** Prevalences of the single item scores

Table S1. UWDRS and GAS for WD Tier 2 (sub)scores depending on gender, KFR, and liver cirrhosis


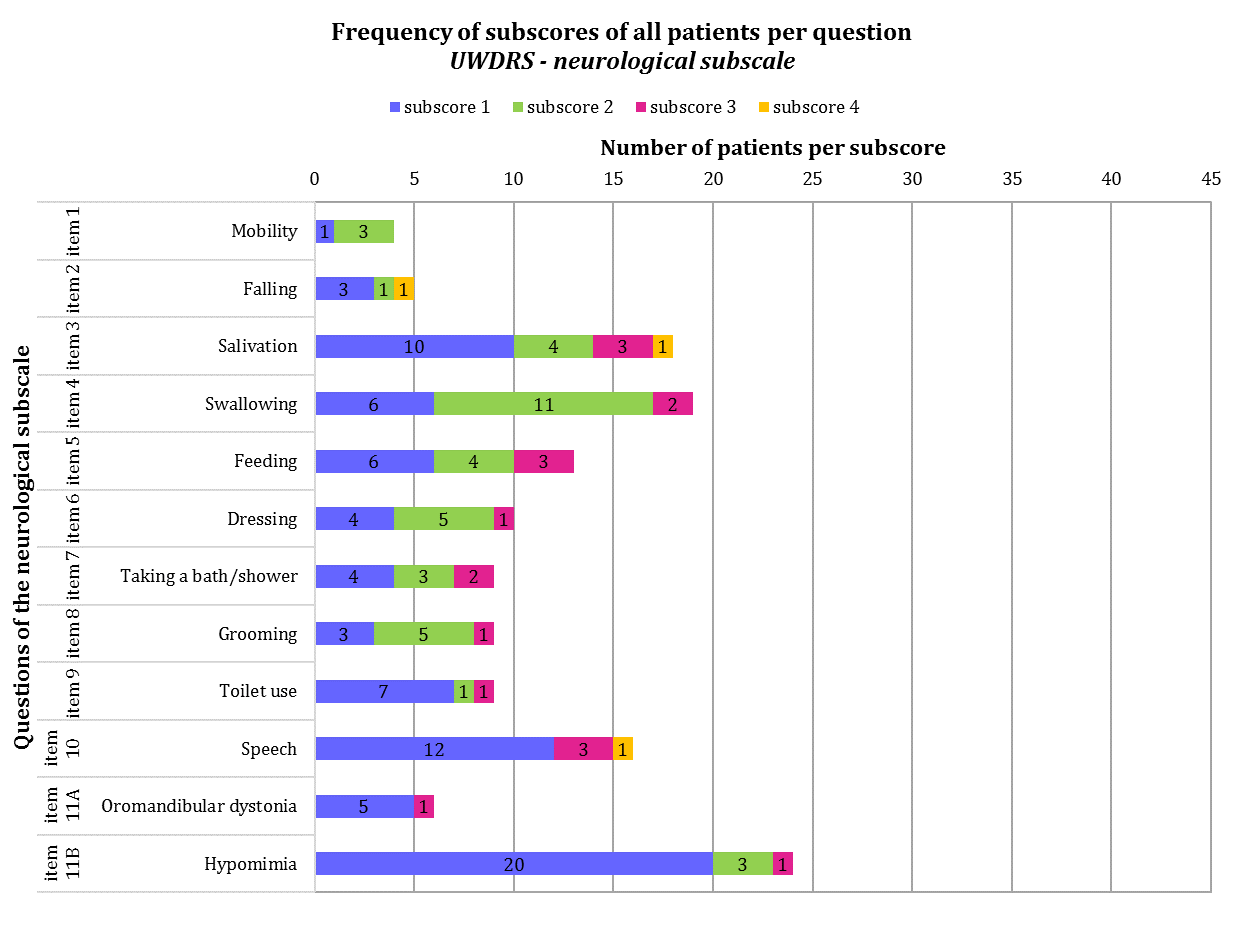


| **Single-item total score correlation** | **Cronbach’s α for omitted item** |
| --- | --- |
| 0.68 | 0.96 |
| 0.49 | 0.96 |
| 0.45 | 0.96 |
| 0.50 | 0.96 |
| 0.77 | 0.96 |
| 0.84 | 0.96 |
| 0.74 | 0.96 |
| 0.85 | 0.96 |
| 0.81 | 0.96 |
| 0.62 | 0.96 |
| 0.35 | 0.96 |
| 0.57 | 0.96 |

| **Single-item total score correlation** | **Cronbach‘s α for omitted item** |
| --- | --- |
| 0.55 | 0.96 |
| 0.45 | 0.96 |
| -0.02 | 0.96 |
| -0.02 | 0.96 |
| 0.41 | 0.96 |
| 0.33 | 0.96 |
| 0.48 | 0.96 |
| 0.51 | 0.96 |
| - | - |
| - | - |
| 0.81 | 0.96 |
| 0.78 | 0.96 |
| 0.69 | 0.96 |
| 0.70 | 0.96 |
|  |  |


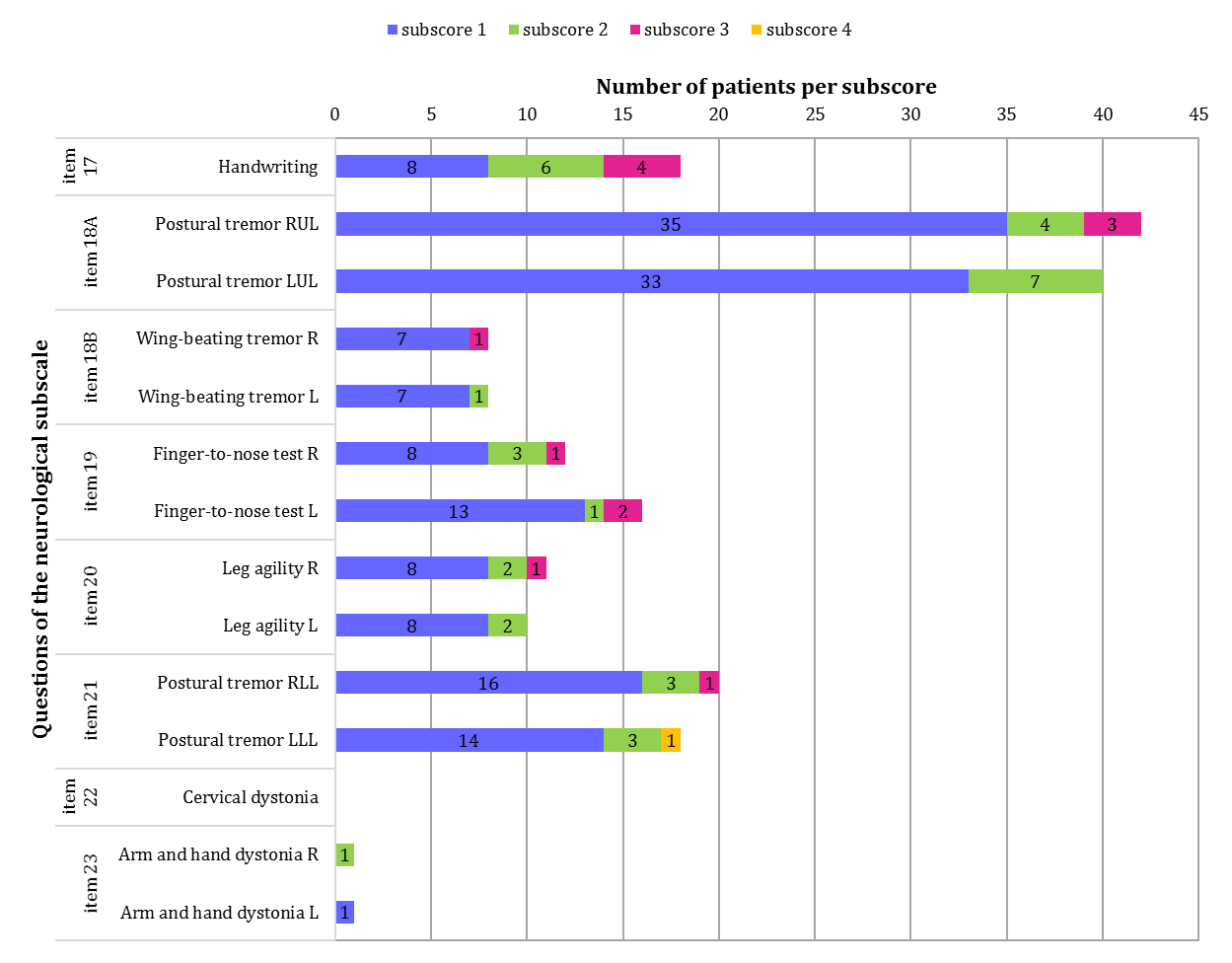


| **Single-item total score correlation** | **Cronbach‘s α for omitted item** |
| --- | --- |
| 0.83 | 0.96 |
| 0.55 | 0.96 |
| 0.38 | 0.96 |
| 0.49 | 0.96 |
| 0.63 | 0.96 |
| 0.66 | 0.96 |
| 0.65 | 0.96 |
| 0.55 | 0.96 |
| 0.68 | 0.96 |
| 0.62 | 0.96 |
| 0.61 | 0.96 |
| - | - |
| 0.24 | 0.96 |
| 0.25 | 0.96 |


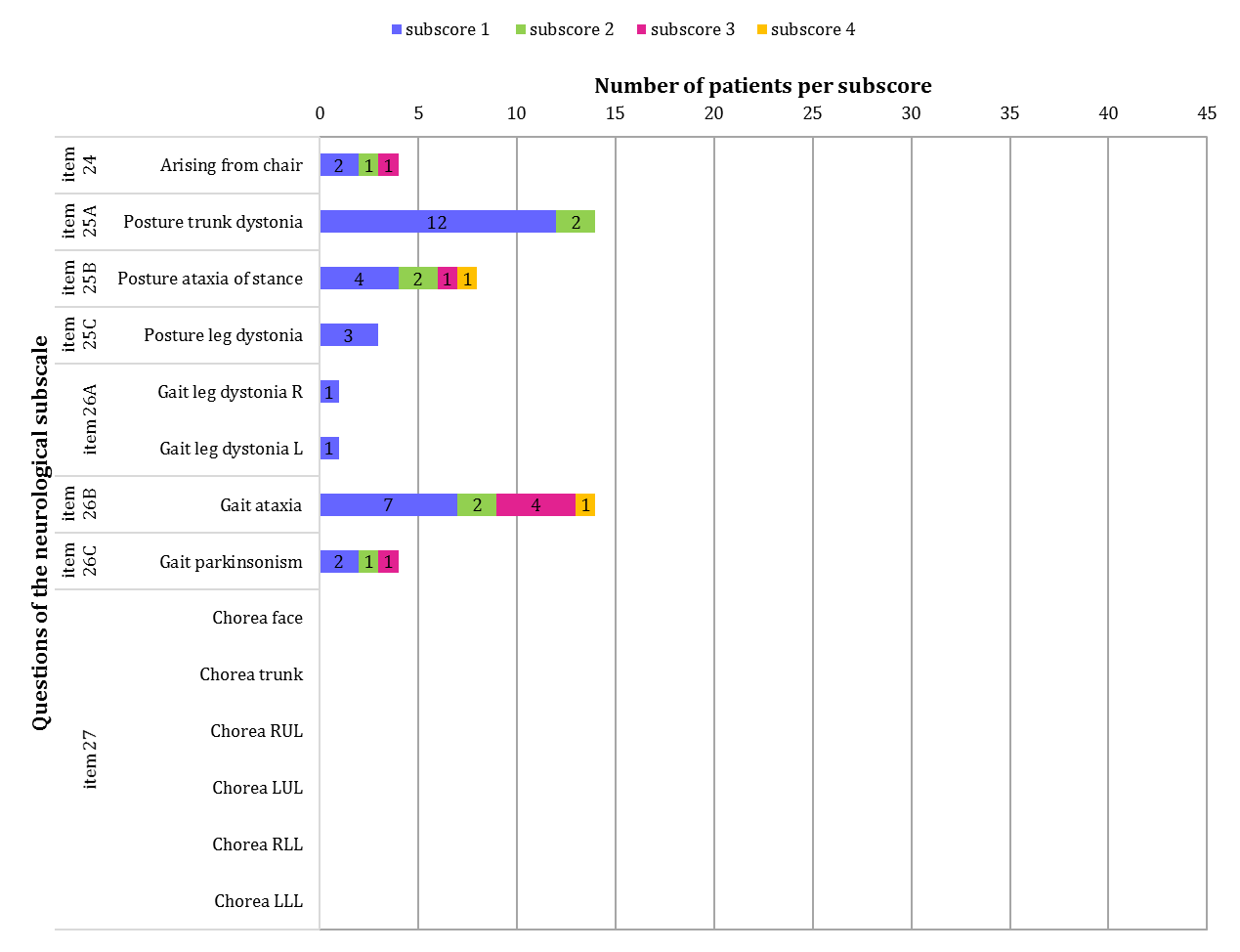


| **Single-item total score correlation** | **Cronbach‘s α for omitted item** |
| --- | --- |
| 0.63 | 0.96 |
| 0.57 | 0.96 |
| 0.76 | 0.96 |
| 0.68 | 0.96 |
| 0.29 | 0.96 |
| 0.29 | 0.96 |
| 0.81 | 0.96 |
| 0.71 | 0.96 |
| - | - |
| - | - |
| - | - |
| - | - |
| - | - |
| - | - |


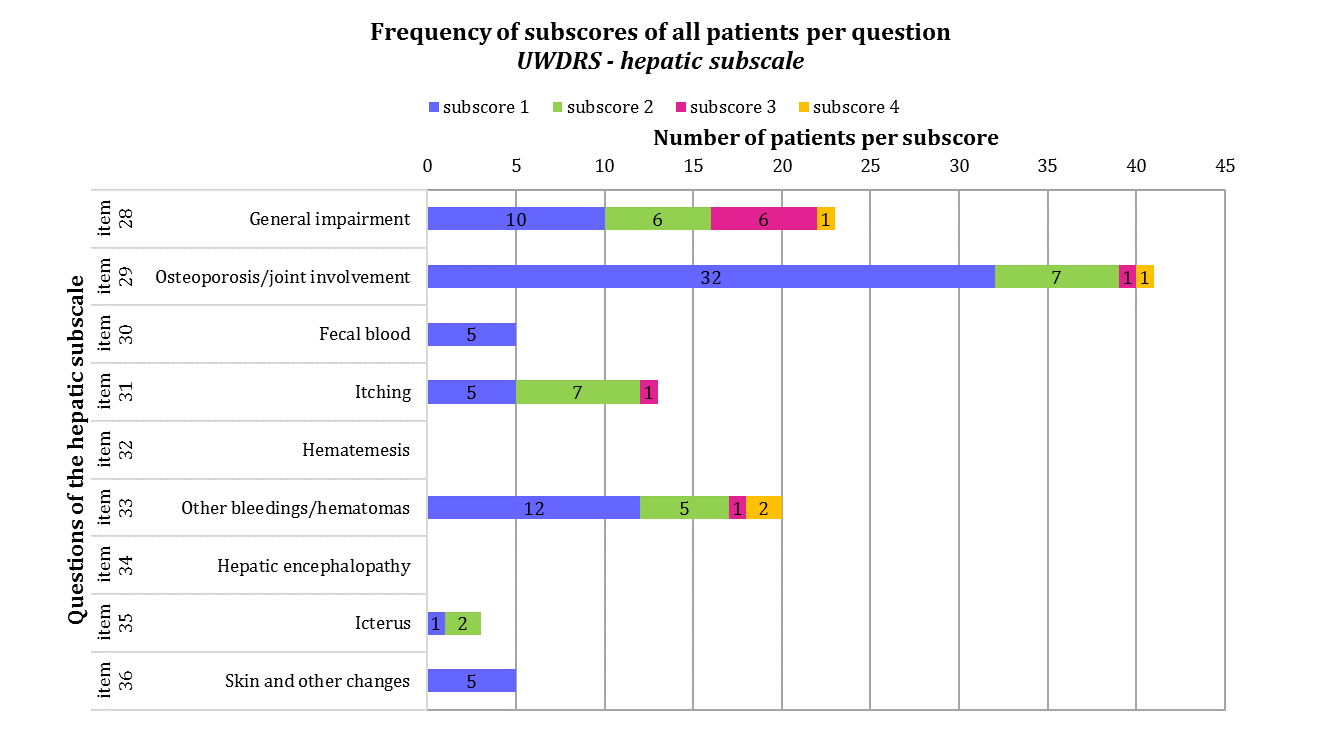


| **Single-item total score correlation** | **Cronbach‘s α for omitted item** |
| --- | --- |
| 0.41 | 0.62 |
| 0.35 | 0.62 |
| 0.14 | 0.66* |
| 0.52 | 0.56 |
| - | - |
| 0.49 | 0.57 |
| - | - |
| 0.39 | 0.62 |
| 0.49 | 0.62 |


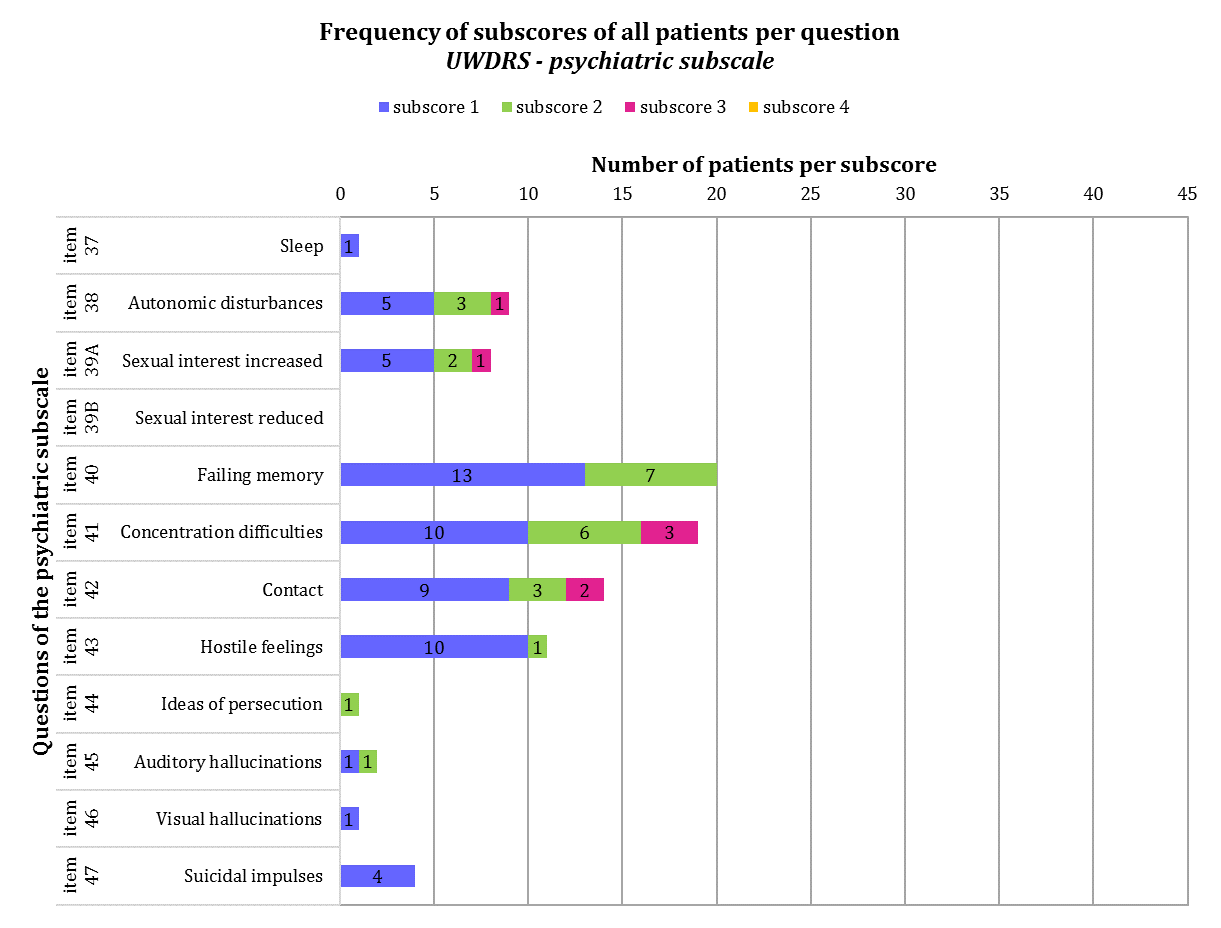


| **Single-item total score correlation** | **Cronbach‘s α for omitted item** |
| --- | --- |
| 0.35 | 0.83 |
| 0.54 | 0.82 |
| 0.31 | 0.84* |
| - | - |
| 0.59 | 0.82 |
| 0.58 | 0.82 |
| 0.55 | 0.82 |
| 0.36 | 0.83 |
| 0.71 | 0.82 |
| 0.65 | 0.82 |
| 0.73 | 0.83 |
| 0.65 | 0.82 |


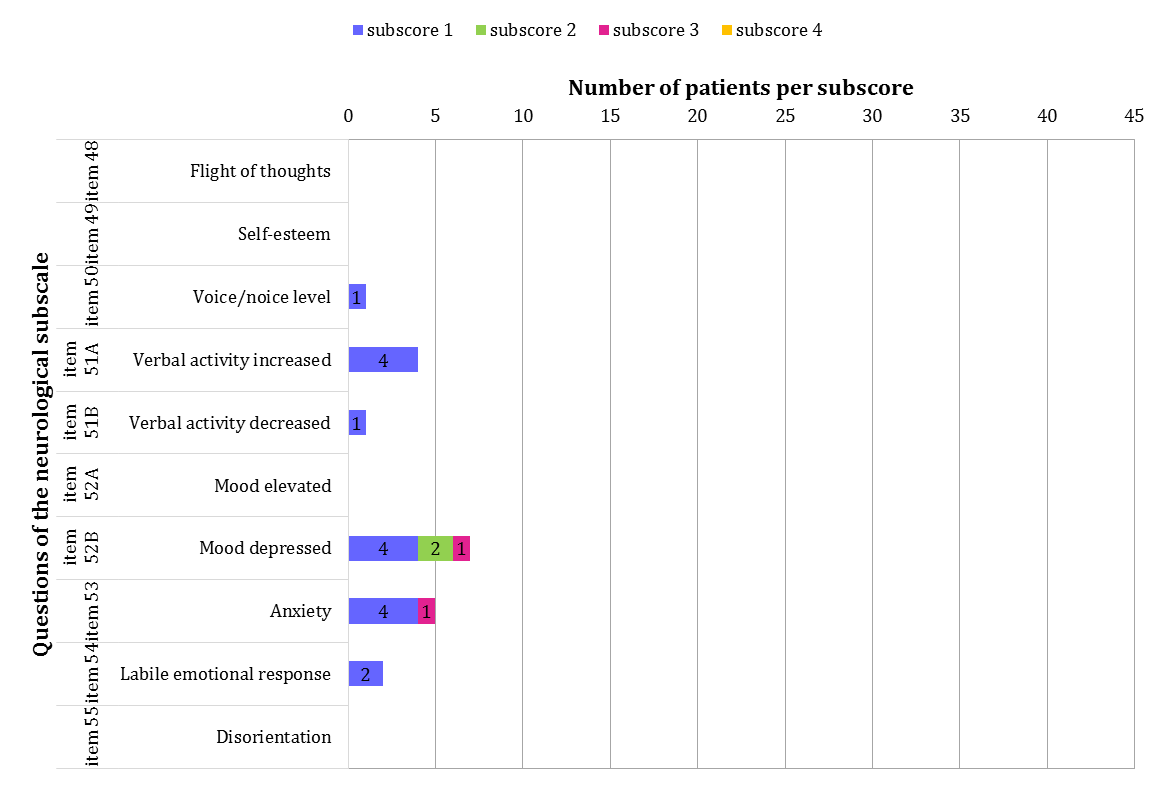


| **Single-item total score correlation** | **Cronbach‘s α for omitted item** |
| --- | --- |
| - | - |
| - | - |
| 0.27 | 0.84* |
| 0.10 | 0.94* |
| - | - |
| 0.62 | 0.84* |
| 0.62 | 0.82 |
| 0.49 | 0.83 |
| - | - |


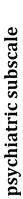

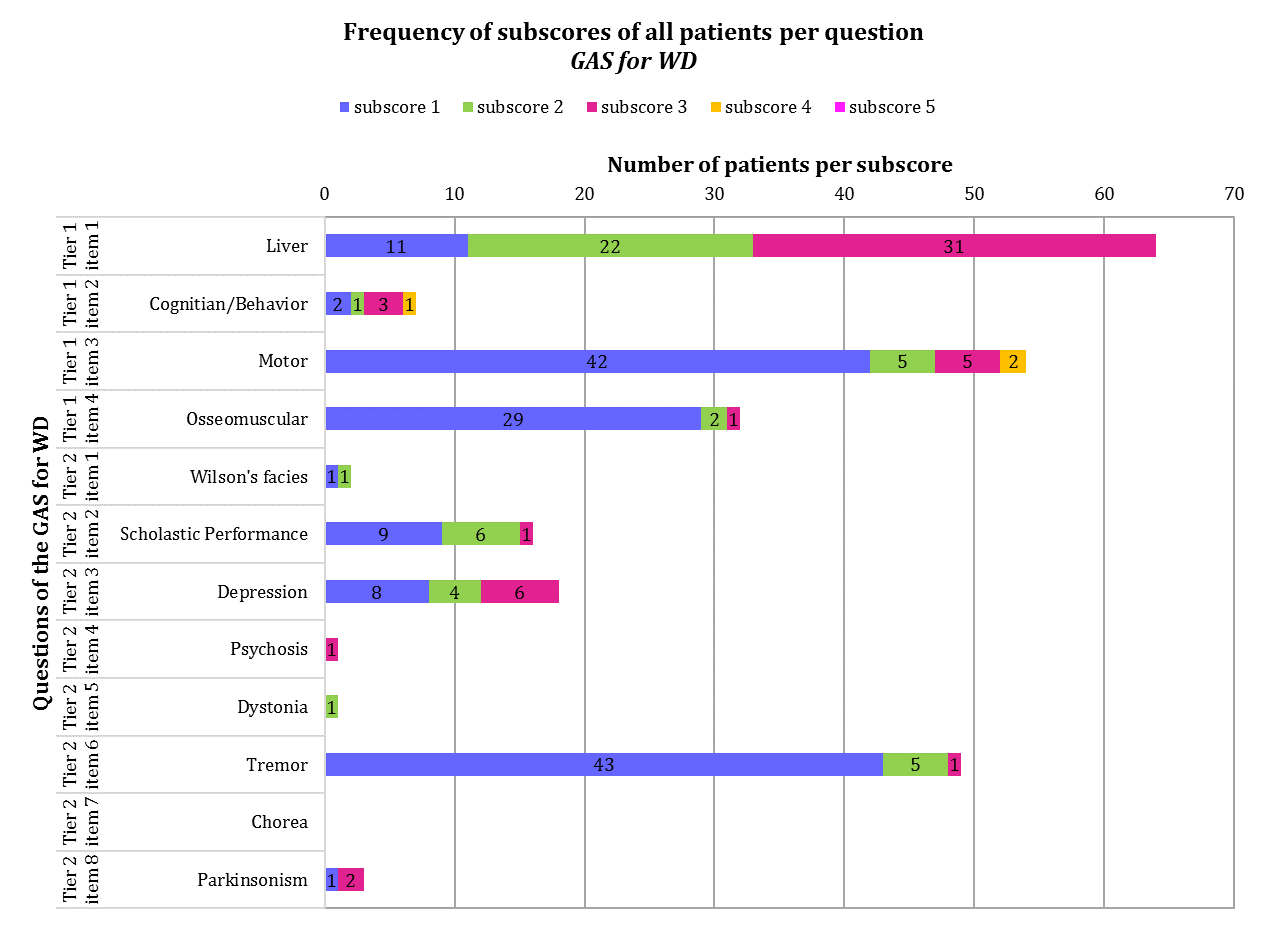


| **Single-item total score correlation** | **Cronbach‘s α for omitted item** |
| --- | --- |
|  |  |
|  |  |
|  |  |
|  |  |
| 0.48 | 0.82 |
| 0.65 | 0.79 |
| 0.57 | 0.80 |
| 0.26 | 0.82 |
| 0.45 | 0.82 |
| 0.29 | 0.82 |
| - | - |
| 0.41 | 0.81 |


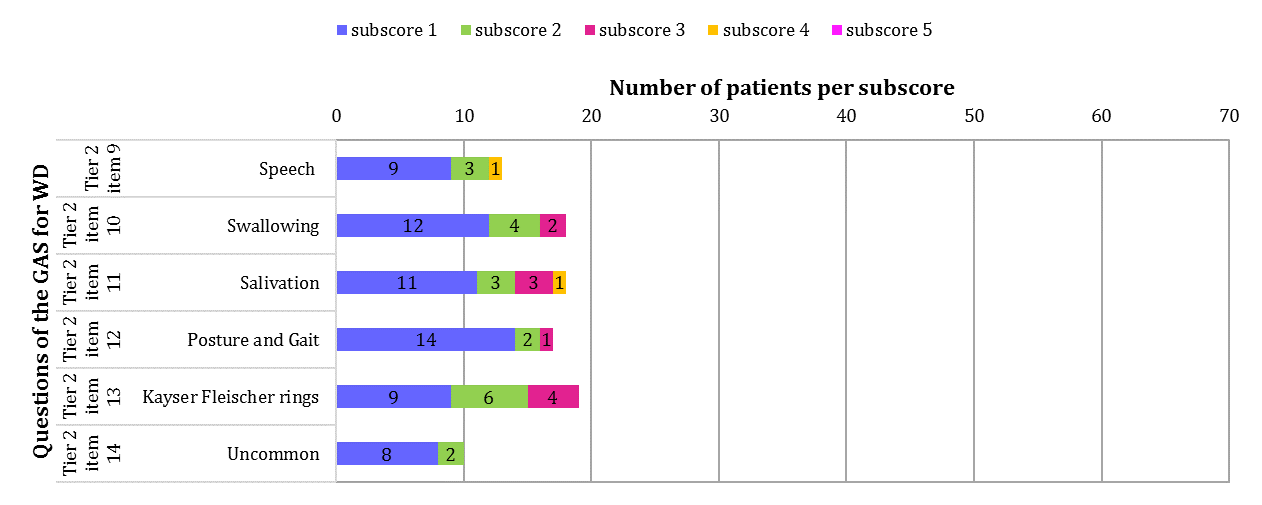


| **Single-item total score correlation** | **Cronbach‘s α for omitted item** |
| --- | --- |
| 0.66 | 0.79 |
| 0.73 | 0.79 |
| 0.62 | 0.80 |
| 0.51 | 0.81 |
| 0.22 | 0.84* |
| 0.45 | 0.81 |

Table S1. UWDRS and GAS for WD Tier 2 (sub)scores depending on gender, KFR, and liver cirrhosis

|  | Total score  median (range) | | P value |
| --- | --- | --- | --- |
|  | **Gender** | |  |
| UWDRS total score  UWDRS neurological subscore  UWDRS hepatic subscore  UWDRS psychiatric subscore  GAS for WD Tier 2 score | **Female**  10 (0-97)  5 (0-66)  3 (0-13)  1 (0-26)  3 (0-17) | **Male**  9.5 (1-76)  5 (1-74)  1 (0-8)  1 (0-9)  2.5 (0-24) | 0.844  0.598  0.004*  0.562  0.775 |
|  | **KFR at assessment** | |  |
| UWDRS total score  UWDRS neurological subscore  UWDRS hepatic subscore  UWDRS psychiatric subscore  GAS for WD Tier 2 score | **Yes**  20 (2-55)  11 (1-49)  2 (0-6)  2 (0-7)  4 (2-24) | **No**  8.5 (0-97)  4 (0-74)  1.5 (0-13)  1 (0-26)  1.5 (0-17) | 0.016*  0.004*  0.763  0.453  <0.001* |
|  | **Liver cirrhosis at the time of diagnosis** | |  |
| UWDRS total score  UWDRS neurological subscore  UWDRS hepatic subscore  UWDRS psychiatric subscore  GAS for WD Tier 2 score | **Yes**  14 (0-55)  7 (0-49)  4 (0-13)  3 (0-12)  3 (0-24) | **No**  9 (1-97)  5 (1-74)  1 (0-7)  1 (0-26)  2.5 (0-17) | 0.198  0.422  0.078  0.136  0.702 |

*, P value statistically significant
